# Near-Infrared Reflectance Spectroscopy for Predicting the Phospholipid Fraction and the Total Fatty Acid Composition of Freeze-Dried Beef

**DOI:** 10.3390/s21124230

**Published:** 2021-06-20

**Authors:** Guillermo Ripoll, Sebastiana Failla, Begoña Panea, Jean-François Hocquette, Susana Dunner, Jose Luis Olleta, Mette Christensen, Per Ertbjerg, Ian Richardson, Michela Contò, Pere Albertí, Carlos Sañudo, John L. Williams

**Affiliations:** 1 Centro de Investigación y Tecnología Agroalimentaria de Aragón (CITA), Avda. Montañana, 930, 50059 Zaragoza, Spain; bpanea@cita-aragon.es (B.P.); perealbertilasalle@gmail.com (P.A.); 2 Instituto Agroalimentario de Aragón—IA2 (CITA-Universidad de Zaragoza), Avda. Miguel Servet, 177, 50013 Zaragoza, Spain; olleta@unizar.es; 3 CREA, Consiglio per la Ricerca in Agricoltura e L’analisi dell’Economia Agraria, 00015 Monterotondo, Italy; sebastiana.failla@crea.gov.it (S.F.); michela.conto@creagov.onmicrosoft.com (M.C.); 4 INRAE, Institut National de Recherche pour l’Agriculture, l’alimentation et l’Environnement-VetAgro Sup, UMR1213, Unité de Recherches sur les Herbivores, Theix, F-63122 Saint-Genès Champanelle, France; jean-francois.hocquette@inrae.fr; 5 Departamento de Producción Animal, Facultad de Veterinaria, Universidad Complutense, 28040 Madrid, Spain; dunner@vet.ucm.es; 6 Departamento de Producción Animal, Universidad de Zaragoza, Avda. Miguel Servet, 177, 50013 Zaragoza, Spain; csanudoastiz@gmail.com; 7 Frontmatec Smoerum A/S, Hassellunden 9, DK-2765 Smoerum, Denmark; mch@frontmatec.com; 8 Department of Food and Nutrition, University of Helsinki, 00014 Helsinki, Finland; per.ertbjerg@helsinki.fi; 9 Bristol Veterinary School, Faculty of Health Sciences, Langford, Bristol BS40 5DU, UK; isnandimelda@gmail.com; 10 Davies Research Centre, School of Animal and Veterinary Sciences, University of Adelaide, Roseworthy, SA 5371, Australia; john.williams01@adelaide.edu.au; 11 Department of Animal Science, Food and Nutrition, Università Cattolica del Sacro Cuore, via Emilia Parmense, 84, 29122 Piacenza, Italy

**Keywords:** NIRS, muscle, bovine, chemometrics, MUFA, PUFA, SFA

## Abstract

Research on fatty acids (FA) is important because their intake is related to human health. NIRS can be a useful tool to estimate the FA of beef but due to the high moisture and the high absorbance of water makes it difficult to calibrate the analyses. This work evaluated near-infrared reflectance spectroscopy as a tool to assess the total fatty acid composition and the phospholipid fraction of fatty acids of beef using freeze-dried meat. An average of 22 unrelated pure breed young bulls from 15 European breeds were reared on a common concentrate-based diet. A total of 332 longissimus thoracis steaks were analysed for fatty acid composition and a freeze-dried sample was subjected to near-infrared spectral analysis. 220 samples (67%) were used as a calibration set with the remaining 110 (33%) being used for validation of the models obtained. There was a large variation in the total FA concentration across the animals giving a good data set for the analysis and whilst the coefficient of variation was nearly 68% for the monounsaturated FA it was only 27% for the polyunsaturated fatty acids (PUFA). PLS method was used to develop the prediction models. The models for the phospholipid fraction had a low R^2^p and high standard error, while models for neutral lipid had the best performance, in general. It was not possible to obtain a good prediction of many individual PUFA concentrations being present at low concentrations and less variable than other FA. The best models were developed for Total FA, saturated FA, 9c18:1 and 16:1 with R^2^_p_ greater than 0.76. This study indicates that NIRS is a feasible and useful tool for screening purposes and it has the potential to predict most of the FA of freeze-dried beef.

## 1. Introduction

Near-infrared spectroscopy has been used for many years to measure the chemical composition of raw materials in the agri-food industry because it is a rapid, clean and accurate tool. The meat industry routinely uses infrared spectroscopy to analyse the chemical composition of meats [1,2,3,4,5,6]. 

Research on the fatty acid (FA) composition of meats has been growing in response to consumer concerns about their healthiness. It is widely known that the intake of monounsaturated FA (MUFA) and polyunsaturated FA (PUFA), mainly n-3 FA, reduces the prevalence of coronary heart disease and cholesterol levels [7] and other inflammatory and immune disorders [8]. Conversely, high intakes of saturated fatty acids (SFA) is associated with increased susceptibility to heart attacks due to the formation of blood clots [9], although that relationship remains unclear [10]. However, whilst high concentrations of PUFA in meat may be nutritionally desirable [11], in the absence of adequate concentrations of antioxidant, it can increase meat colour intensity (saturation) and fat oxidation resulting in poor sensory quality [12,13]. The FA composition of meat is usually determined by gas chromatography. When this method is optimized and long columns are used it allows many fatty acids to be quantified. However, gas chromatography is expensive, slow and uses dangerous chemicals. NIR spectroscopy is fast, cheap and clean and is useful in estimating multiple characteristics at the same time. It is not used widely because not all minor FA are accurately determined [14,15,16,17], partly because of the high water content of meat that absorbs more infrared light than the solutes [18,19], and as a result, calibration often fails. The absorbance of materials with high moisture content is temperature dependent [20] which also makes it difficult to calibrate the analyses. Consequently, removing water from materials before NIRS analysis is likely to improve the quantification of certain substances [19,21,22,23]. Freeze drying meat prior to analysis has been shown to improve the determination of minor fatty acids such as individual FA [17]. In addition to improving performance compared with raw meat because water absorption bands are reduced, freeze-drying also concentrates substances in beef around fourfold [24]. NIRS can determine fatty acid profiles of phospholipid and neutral lipid fractions independently. Using this technique SFA and MUFA concentrations in the neutral fraction have been shown to be higher than in the phospholipid fraction while PUFA is higher in the phospholipid fraction than the neutral lipid fraction [25]. The ability to study the fatty acids of neutral and phospholipid fractions is of interest because phospholipids are the building blocks of the cell membrane. The fatty acid composition of the membrane is mainly controlled by the genes involved in fatty acid metabolism, whilst the fatty acid composition of neutral lipid is influenced by the diet [26,27]. The positive impact of dietary phospholipids on human health is well established [28] and in addition, phospholipids contribute to the flavour of meat together with the Maillard reactions [26,29].

This work evaluated near-infrared reflectance spectroscopy as a tool to assess the total fatty acid composition and the phospholipid fraction of fatty acids of beef using freeze-dried meat.

## 2. Materials and Methods

### 2.1. Animal and Rearing Conditions 

The care and use of animals were in accordance with the European Union Directive 2010/63 on the protection of animals used for experimental and other scientific purposes [30] because, at the time of the experiment, the member states of the European Union had no obligation to have an Ethics Committee for Animal Experiments. 

A total of 332 unrelated pure breed young bulls from 15 European breeds were reared on commercial farms or in experimental research centres, depending on the experimental facilities of each country in France, Denmark, Italy, Spain and the United Kingdom. A uniform beef management system, representative of those used in European Union countries, was used for all breeds to standardise, as far as possible, the influence of diet, management and rearing systems on meat quality. The breeds and number of animals were: Aberdeen Angus (27), Highland (24), Jersey (25), South Devon (20), Danish Red (24), Holstein (25), Simmental (20), Asturiana de la Montaña (22), Asturiana de los Valles (20), Avileña-Negra Ibérica (22), Pirenaica (20), Marchigiana (22), Piemontese (20), Charolais (21) and Limousin (20). At 9 months of age, all the animals were transferred to the experimental farms, where they were divided into groups of 7 to 8 animals, and fed the standardised diet, which consisted of a concentrate compounded from barley flakes (80 to 84%), soya bean meal (7.5 to 11%) sodium bicarbonate (0.6%) with vitamin supplements (1.5%) and barley straw, fed ad libitum. The energy density ratio ranged from 12.9-13.5 ME/kg DM. The protein content was 160 g Crude Protein/kg DM up to 10 months of age and then decreased to 150 g CP/kg DM to slaughter. Performance, body size and carcass characteristics of the fifteen breeds have been previously reported by Albertí, et al. [31]. 

### 2.2. Sampling and Measurements

At 75% mature bull weight, which was at about 15 months of age, animals were slaughtered as reported in Albertí, et al. [31]. The carcasses were chilled at 4 °C for 24 h. Then, the longissimus thoracis (LT) muscle was excised from the left side of the carcass between the 6th and the 13th ribs and was stored at 2 °C ± 1 °C until 48 h post-mortem. Then, a steak per animal was taken from around the position of the 8th vertebra and split into two pieces. Both pieces were vacuum packed and frozen at −18 °C. One-piece was transported in polystyrene boxes filled with dry ice to the CREA-ZA (Monterotondo, Italy) for NIR analysis, while the other was transported to the University of Bristol (Bristol, United Kingdom) for fatty acid analysis.

### 2.3. Collection of NIR Reflectance Spectra

The intermuscular fat covering of the LT was discarded and the remaining sample was freeze-dried and stored at −70 °C until spectra collection. The sample was homogenized using a meat mincer Moulinex D-56 (Groupe SEB, Écully, France) and kept at room temperature for 1 h before recording the spectra. The minced freeze-dried sample was inserted into a cylindrical quartz glass cup with an internal diameter of 35 mm and a depth of 10 mm. Reflectance spectra were scanned and collected twice per sample with a FOSS NIRSystems 5000 (FOSS NIRSystems Inc., Silver Spring, MD, USA). Spectra were recorded from 10,000 to 4000 cm^−1^ each 2 cm^−1^ interval (1000 to 2500 nm each 0.5 nm interval) and recorded as $\log\left(\frac{1}{R}\right)$. 

### 2.4. Fatty Acid Composition Analysis

The samples for fatty acid determination were stored at –70 °C until analysis. After thawing in tap water, the muscle was blended and the lipids were extracted from 10 g samples using chloroform:methanol (2:1, *v/v*) [32], separated into neutral lipid (NL) and phospholipid (PL) fractions, using silicic acid chromatography (Isolute Si, Jones Chromatography, Hengoed, Glamorgan, UK) and methylated as described in Scollan, et al. [33] using a solution of diazomethane in diethyl ether. Total lipid content was taken as the sum of the neutral lipid and phospholipid fractions. Samples were analysed by gas chromatography by injection in the split mode, 70:1, onto a CP Sil 88, 50 m × 0.25 mm fatty acid methyl esters (FAME) column (Chrompack UK Ltd., London, UK) with helium as the carrier gas. The individual peaks of each FA were identified and quantified as described in detail by Scollan, et al. [33]. Only the major fatty acids were reported, which represented over 90% of the total FA present. 

### 2.5. Chemometrics

The samples from each breed were assigned to a Calibration or a Validation set randomly. Random numbers between 0 and 1 were generated using MS-Excel and they were assigned to each sample. Therefore, samples with a number lower than 0.67 were assigned to the Calibration set and the remaining samples were assigned to Validation set. Hence, the Calibration set comprised 222 samples (67%), while the Validation set had 110 samples (33%). Mathematical treatments and pre-treatments such as scatter correction and derivatives, as well as gap and smooth segments, were investigated. Forty-eight 48 optimal models were developed including FA of the total and phospholipid fraction and groups of FA. The number of factors for each model were selected to optimize the R^2^. Spectral ranges and individual wavelengths were selected according to the loadings and regression coefficients of the models, and then tested to obtain the best calibration model. Because many of the data points in the spectrum were highly co-linear, they were compressed using few factors [34] to derive the calibration equation. Compression was carried out using Partial Least Squares. The performance of the different predictive models obtained were determined from calibration and validation. The standard errors of calibration (SEC) and validation (SEP) sets, the coefficients of determination (R^2^_c_ and R^2^_p_) of calibration and validation, respectively the residual predictive value (RPD) and Consistency were used to test the accuracy of the calibration models [35] and to choose the best model. RPD was calculated as ($RPD=\frac{SD}{SEP}$), where SD is the standard deviation of the laboratory (SD). Consistency was calculated as ($C=\frac{SEC}{SEP}\cdot 100$) and expressed as a percentage [36]. The Hotelling statistic (H statistics) was calculated and samples with an H statistic greater than 10 were defined as outliers. When outliers were eliminated from the calibration set the model improved. Chemometrics and spectral data management were carried out with Unscrambler X (Camo Software AS, Norway). Calibration equations were derived for the phospholipid fraction of FA and total FA but not the neutral FA as these can be calculated by the difference between total and phospholipid fraction.

## 3. Results and Discussion

### 3.1. Sample Composition

Twenty fatty acids were detected, and 4 FA groups were calculated. Table 1 and Table 2 shows the descriptive statistics of the phospholipid fraction of FA and total FA, respectively. Means of total FA were higher than means of the FA of phospholipid fraction but differences were much higher for SFA and MUFA than PUFA. In general, total FA was more variable than the phospholipid fraction, as expected, because the different carcass fatness of the animals used in the study is related to the neutral lipid fraction, while the phospholipid fraction is more constant and less susceptible to differences in bodycomposition [37]. Therefore, the coefficient of variation (CV) of total FA from 11 FA was higher than 60% while only 4 FA had a CV of the phospholipid fraction higher than this value. Moreover, total SFA, total MUFA and total FA also had a CV above 65% while the phospholipid fraction had a CV below 30%. The total FA (Table 2) ranged from 2.7% to 10.9%. 

In the calibration set, neutral and phospholipid fractions were 81.7% and 18.3%, respectively, in agreement with MacKintosh, et al. [37]. The phospholipid fraction contained much more PUFA than the neutral lipid (13.5% of SFA, 9.6% of MUFA and 74.1% of PUFA) while 18:1c9, 18:1t9 and CLA had lower percentages in phospholipid fraction (9.9%, 9.0% and 9.1% respectively) than in neutral lipid, which is in agreement with the findings of Wood, et al. [38] and MacKintosh, et al. [37]. Conversely, the percentages of FA with 20 or more carbons were greater in the phospholipid fraction than in neutral lipid. Therefore, the percentages of 20:3n-6, 20:4n-6, 20:5n-3, 22:4n-6, 22:5n-3 and 22:6n-3 in the phospholipid fraction were 92.3%, 98.1%, 96.2%, 93.5%, 95.4% and 95.6%, respectively [9].

Variations in the FA profiles from the 15 breeds used in the current study are representative of the variation present in European beef FA. The 15 breeds included different cattle types, milk, meat and dual purpose. The individual profiles and different lipid ratios of the 15 European cattle breeds included in the study are given in Sevane, et al. [39] and their correlations with sensory traits, such as flavour, texture or juiciness in Sevane, et al. [40]. However, as the animals were fed a standardized diet the variations were lower than if they had been fed different diets, especially in PUFA [27]. Andueza, et al. [17] noted that the variation in FA in cattle is limited because of the biohydrogenation of ruminants during the digestion process. The goodness of calibration models relies on the variability of samples in the data set used to develop the prediction models [14,24], but increasing the variability in the data does not always increase the accuracy of the calibration. Khan, et al. [41] used meat from four different species to increase the variability and showed that the calibration for the chemical composition could not be improved. The total amount of fat influences the fatty acid composition. Therefore, increasing the age of animals and fattening could increase the variation in FA, but this does not help for the phospholipid fraction which remains constant even though the total lipids increase [42]. 

Most of the recent studies on beef used absolute values to develop the calibration models, and have achieved good statistical results [14,43] although other authors still express the results as a percentage of total fatty acids [24]. The absorbance varies linearly with the parameter concentration, not the relative ratio, therefore using absolute content gives a better calibration than the use of percentages or relative amounts [44].

The use of freeze-dried samples increases the relative concentration of fatty acids and so improves the results [24]. In addition, freeze-drying reduces the water absorption, improving the resolution of the absorption spectra for muscle. Freeze-drying also has the advantage of fine grinding, which is expected to improve the calibration [45], because it has been demonstrated that mincing meat prior to analysis gives better results than intact meat [46]. As intact muscle fibres and myofibrils tend to conduct NIR light by absorbing more energy [47]. Expressing the results as an absolute value indicates the nutritional value of meat, but differences in the treatment of the data and the way results are expressed make it difficult to compare results among studies.

### 3.2. Spectral Characteristics

**Figure 1:**
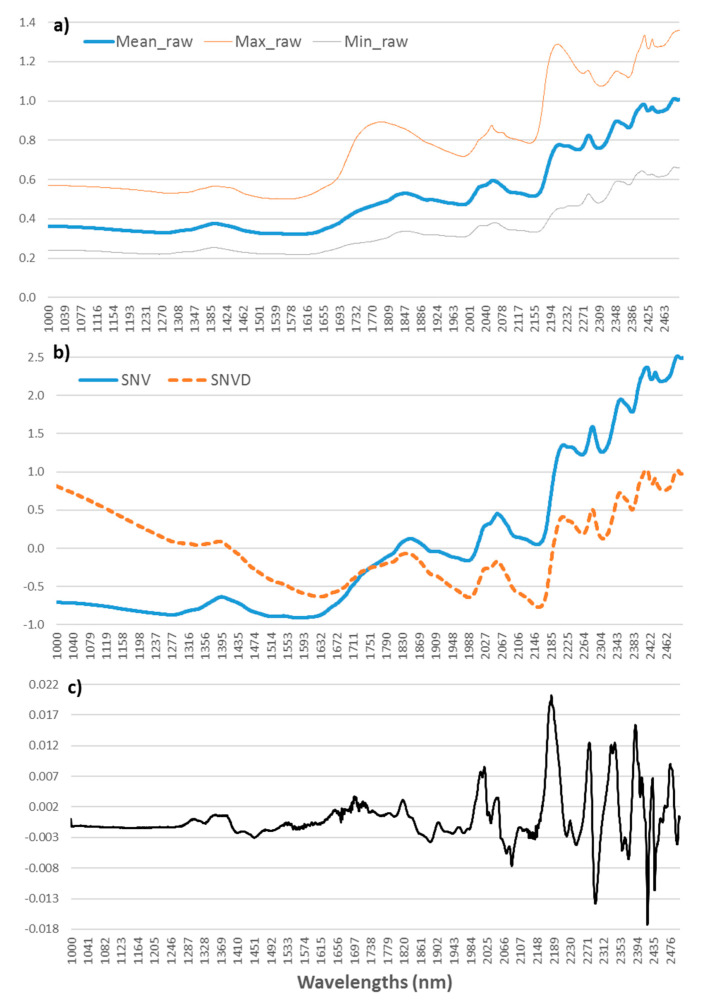
(**a**) Average (bold line), maximum and minimum (thin lines) of raw NIR spectra. (**b**) Standard normal variate (SNV) and standard normal variate plus detrending (SNVD) pre-treatments. (**c**) SNVD and first-order derivative pre-treatments. Spectra were recorded as log(1/R).

The mean spectrum for the 15 breeds started with values of 0.4 (minimum value above 0.2) which was sustained until 1600 nm and finished at 2500 nm with values of 1.0 and a maximum of almost 1.4 (Figure 1). 

Values of absorbance and the shape of the spectrum are similar to the spectrum of freeze-dried beef reported by Andueza, et al. [17]. The absorbance for freeze-dried beef was lower than the absorbance of fresh beef [14,17,48], broiler breast [48] and pork [49] but the shape of the spectrum is similar among all the meats. The low absorbance values in the ranges 1440–1470 nm and 1920–1960 nm are due to the absence of water [50]. Giaretta, et al. [24] compared spectra from fresh and freeze-dried beef and reported similar values to ours at 1000 nm but much higher values at 2500 nm. In addition, these authors found more sharp peaks around 1700 nm and also in the C–H resonance region (2200–2500 nm). Within that latter band, Zhou, et al. [48] reported absorbance peaks, specifically 2310 nm and 2348 nm, which are related to lipids. In the present study, this region had subtle peaks which were highlighted with the use of scattering corrections and derivatives (Figure 1a,b). In that region, we also found the highest regression coefficients for the prediction of total FA and many individual FA (data not shown), which is in agreement with the findings of Prieto, et al. [51].

### 3.3. Prediction Models

The spectral pre-treatments and factors used to develop calibrations of phospholipids and total fatty acids used in this study are shown in Table 3 and Table 4. 

**Figure 2:**
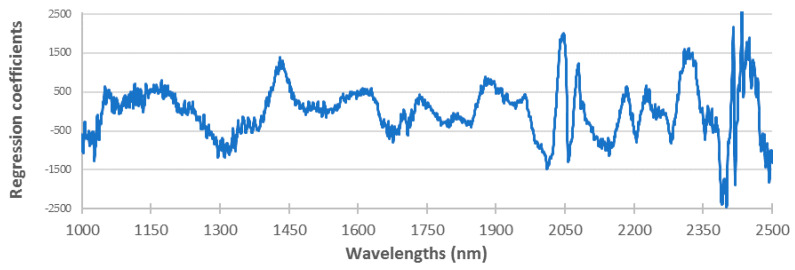
Regression coefficients of each wavelength for the model of total fatty acids.

The offset baseline correction was used in 62.5% of the prediction models for the phospholipid fraction, while it was only used in the 8.3% of the prediction models for the total fraction of fatty acids. Area normalization was used in 50% of the prediction models for the phospholipid fraction and 12.5% of the prediction models for the total fraction of FA. The extended multiplicative scatter correction, SNV and SNVD were used in most of the calibrations of phospholipids (58.3%) but it was only required for 33.3% of calibrations of total fat fraction, mainly SNVD. The Savitzky–Golay derivative of first order with the second polynomial order and a smoothing gap of 3 or 5 were used in 15 calibrations while a Norris gap first-order derivative with large gap sizes (from 11 to 27) was useful to develop 6 calibrations. The most useful mathematical treatments were SNVD with or without first-order derivatives although many optimal models were developed without mathematical treatment. Indeed, the best prediction models of total FA, SFA, 9c18:1 and 16:1 used offset baseline correction, non-treatment, first-order Norris-Gap derivative and non-treatment, respectively. Figure 2 shows the regression coefficients of the model of total fatty acids.

There are no studies focused on the prediction of FA phospholipids, so the total FA fraction will be discussed here. The best statistical results have been found by applying SNV (with or without detrend) with the second-order derivative for most of the FA [49,52,53]. SNVD treatment reduced multicollinearity and the deleterious effects of a baseline shift and curvature while derivatives increase the resolution of peaks and reduce scattering [52]. However, the use of finely ground freeze-dried samples, means that for many calibrations pre-treatments are not needed, which has also been reported by Andueza, et al. [17].

Calibration and validation statistics of the models are shown in Table 5 and Table 6. Some authors [49] reported calibrations for C16:0 and C18:0 with R^2^ of 0.66 and 0.71, respectively, in pork using the SNVD and second-order derivatives, while Sierra, et al. [53] and Cecchinato, et al. [54] found that for beef C16:0 and C18:0 FA had an R^2^ of 0.8 and 0.7, respectively. These values were similar to those using lamb and beef mixed together [14] and for rabbit meat [55]. An R^2^ equal to or greater than 0.9. has been reported for freeze-dried beef [17] and a high R^2^ for predicting C16:0 and C18:0 has also been found for broiler breast using SNVD and first-order derivatives [48]. Therefore, most of the authors reported similar or slightly better results than our results when using beef, pork and lamb with similar mathematical pre-treatments such as SNVD.

Conjugated linoleic acid (CLA) describes a group of 18-carbon fatty acids with two conjugated double bonds. These isomers, of which c9,t11 and t10,c12 are the predominant members in beef, are beneficial for human health [56]. Some authors reported models that are for the entire CLA group, others report models for individual components, in this case, c9t11 and other isomers are used because major CLA isomers can coelute during GC analysis [57]. Hence, the comparison of results is not easy. The model we used (MSC, 1^st^ derivative and R^2^_p_ = 0.62) is consistent with those described in the literature because most used SNVD together 1st or 2nd derivative [14,17,53]. Prieto, et al. [51] used finely ground beef to predict several groups of CLA isomers with R^2^_c_ ranging from 0.77 to 0.84, confirming that the finer the grinding, the higher the accuracy [47]. Finally, other authors did not report CLA [24,50,58].

Most FA with more than 19 carbons were not well predicted in our study, which has also been found by other authors [14,17,48,53,55]. Therefore, these are often not reported and published data tend to focus on the main groups of FA [24,50,52,58]. Most authors reported worse statistics for PUFA than for SFA and MUFA [14,48,53,55,59]. This could be explained because long-chain PUFA are mainly located in the membrane phospholipids which are quite constant because they are controlled by a complex enzymatic system, providing low variability among animals and have relatively low concentrations [58].

**Figure 3:**
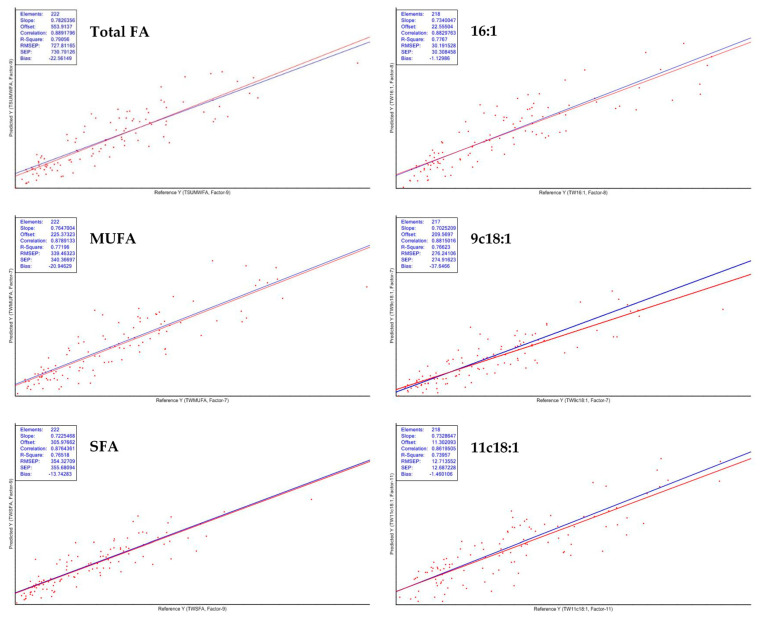
Scatter plots of models of 16:1, 11c18:1, total FA, SFA and MUFA of the total fraction.

The models developed for the phospholipid fraction of the FA had RPD lower than 2 being useful just for screening purposes. However, some models for the estimation of FA were adequate for analytical purposes such as 14:0, 16:1, 11c18:1, total FA, SFA and MUFA. The other models remained below RPD = 2. The plots of those models are shown in Figure 3. The main weakness of NIRS to predict the FA composition is the inconsistency. While gas chromatography can identify all the FA that are important for meat science, NIRS does not. The reasons for the low-quality calibrations of some FA of meat include low concentration and variability, presence of water, and comparison of intact vs ground meat. The poor performance of NIRS in prediction equations for FA is due to this low variability and because some FA absorbs at the same wavelengths [14,47]. In our data set the range of variability may result in a complex relationship between the spectra and the response variables that are not predicted under a PLS model [59]. Using the NIRS technique to predict fatty acids is hampered by the absorption of light by the C–H bonds in certain wavelengths. 

Therefore, a C–H bond together with a cis bond modifies the absorption at the same wavelengths as a double cis bond [60]. This means that some individual fatty acids are not determined accurately, which could be related to similarities in the NIR absorption spectra among FA [48,53]. Other authors that have studied the PLS method, used in our study, fail when the relationship between spectra and the analyte of interest is non-linear [59,61]. Spectra collected in the reflectance mode are influenced not only by the main components of meat (water, fat, protein, etc.) but also the particle size, which is affected by the sample homogenization method and has to be accounted for using the right mathematical preprocessing [62]. Our results, using spectra from milled freeze-dried meat, suggest that this type of sample requires little or no preprocessing.

## 4. Conclusions

This study indicates that NIRS is a feasible and useful tool for screening purposes and has the potential to predict most of the FA of beef. The use of freeze-dried samples, thus reducing the water absorption bands and increasing the concentration of analytes, improved the accuracy of calibrations. Minimal mathematical pre-treatments were required to obtain good results. Using 15 breeds ensured that there was a large variation in the samples, which enabled us to develop good models. However, these improvements were not enough to achieve good calibrations for the phospholipid fraction, mainly due to the low concentrations of the FA in this fraction.

## Figures and Tables

**Table 1:** Descriptive statistics of the phospholipid fraction of fatty acids (mg/100 g of meat) of calibration and validation sets.

	Calibration Set					Validation Set				
Fatty Acid	Mean	Min	Max	S.D.	C.V.	Mean	Min	Max	S.D.	C.V.
12:0	0.06	0.00	0.53	0.08	133.33	0.09	0.57	1.32	0.16	177.78
14:0	1.64	0.34	6.34	1.13	68.90	1.62	2.49	4.96	1.05	64.81
16:0	64.42	34.05	109.85	14.34	22.26	65.92	64.20	103.23	14.93	22.65
16:0 ald	23.42	3.91	43.46	6.42	27.41	24.00	4.650	40.99	7.27	30.29
16:1	7.36	3.07	19.35	2.33	31.66	7.47	6.17	13.79	2.30	30.79
18:0	52.38	29.16	83.93	8.85	16.90	53.44	69.77	73.38	8.81	16.49
18:0 ald	15.87	3.47	26.64	4.48	28.23	16.64	3.36	31.87	5.47	32.87
18:1 t9	4.39	0.93	14.20	2.75	62.64	4.47	6.95	16.85	2.91	65.10
18:1 c9	76.72	26.06	182.51	24.35	31.74	77.08	53.77	137.56	24.19	31.38
18:1 c11	14.58	7.87	25.92	3.43	23.53	14.79	11.29	26.03	3.69	24.95
18:2 n-6	124.47	62.45	210.43	29.61	23.79	127.88	67.43	199.28	29.67	23.20
20:1	0.57	0.00	1.20	0.22	38.60	0.59	0.30	1.26	0.23	38.98
18:3 n-3	6.37	1.51	22.07	4.35	68.29	6.57	3.58	19.57	4.55	69.25
18:2 9c11tCLA	0.75	0.18	2.49	0.41	54.67	0.77	0.70	2.71	0.45	58.44
20:3 n-6	8.43	4.94	15.00	2.00	23.72	8.44	3.60	13.66	2.22	26.30
20:4 n-6	37.84	20.29	69.55	9.39	24.82	38.63	16.27	69.77	9.69	25.08
20:5 n-3	4.02	1.00	12.07	2.01	50.00	4.20	1.23	10.97	2.27	54.05
22:4 n-6	4.59	1.34	10.40	1.82	39.65	4.67	1.10	10.21	1.94	41.54
22:5 n-3	9.17	4.26	21.13	2.94	32.06	9.35	3.43	20.38	3.30	35.29
22:6 n-3	0.87	0.00	4.85	0.48	55.17	0.85	0.00	2.28	0.40	47.06
Total FA	493.81	270.43	813.78	85.49	17.31	504.13	467.25	726.78	89.74	17.80
SFA	157.80	89.26	252.16	28.679	18.17	161.71	101.33	228.035	31.09	19.23
MUFA	103.61	46.35	228.31	28.18	27.20	104.400	47.82	169.93	28.26	27.07
PUFA	196.51	107.55	319.65	39.61	20.16	201.36	103.90	297.84	40.64	20.18

SD, standard deviation; FA, fatty acid; SFA, saturated fatty acid; MUFA, monounsaturated fatty acid; PUFA, polyunsaturated fatty acid; C.V., coefficient of variation.

**Table 2:** Descriptive statistics of the total fatty acid composition (mg/100 g of meat) of calibration and validation sets.

	Calibration Set					Validation Set				
Fatty Acid	Mean	Min	Max	S.D.	C.V.	Mean	Min	Max	S.D.	C.V.
12:0	2.10	0.00	8.86	1.87	89.05	2.06	0.06	8.69	1.80	87.38
14:0	73.63	0.87	313.13	58.97	80.09	71.57	2.49	256.53	55.15	77.06
16:0	651.20	51.20	2878.25	480.58	73.80	639.85	64.20	2420.91	442.30	69.13
16:0 ald	23.42	3.91	43.46	6.42	27.41	24.00	4.65	40.99	7.27	30.29
16:1	92.50	5.55	414.79	73.77	79.75	89.04	6.17	266.67	64.18	72.08
18:0	401.08	50.04	1741.09	254.19	63.38	398.22	69.77	1497.58	237.68	59.69
18:0 ald	15.87	3.47	26.64	4.48	28.23	16.64	3.36	31.87	5.47	32.87
18:1 t9	77.48	3.47	625.86	76.14	98.27	75.20	6.95	363.93	68.72	91.38
18:1 c9	854.17	31.86	4125.90	658.56	77.10	831.04	53.77	2866.55	573.11	68.96
18:1 c11	49.02	11.24	191.83	29.18	59.53	47.77	11.29	119.64	25.02	52.38
18:2 n-6	183.30	83.15	500.18	62.86	34.29	183.30	67.43	314.36	54.76	29.87
20:1	4.03	0.27	22.16	3.28	81.39	3.77	0.30	13.58	2.58	68.44
18:3 n-3	15.16	2.70	69.20	12.27	80.94	14.99	3.58	48.81	11.81	78.79
18:2 9c11t CLA	8.14	0.45	43.18	6.39	78.50	7.84	0.70	27.41	5.26	67.09
20:3 n-6	9.14	5.15	16.12	2.48	27.13	9.11	3.60	16.29	2.67	29.31
20:4 n-6	38.56	20.29	71.23	9.60	24.90	39.32	16.27	70.47	10.01	25.46
20:5 n-3	4.18	1.00	15.56	2.18	52.15	4.35	1.23	11.41	2.37	54.48
22:4 n-6	4.91	1.34	11.33	2.15	43.79	4.96	1.10	11.70	2.26	45.56
22:5 n-3	9.61	4.26	21.85	3.11	32.36	9.82	3.43	20.65	3.47	35.34
22:6 n-3	0.91	0.00	4.95	0.61	67.03	0.89	0.00	5.56	0.60	67.42
Total FA	2701.26	452.75	10922.01	1780.47	65.91	2652.12	467.25	8701.06	1596.84	60.21
SFA	1167.30	133.90	4981.92	790.24	67.70	1152.34	157.41	4182.02	734.39	63.73
MUFA	1077.19	54.47	4916.56	818.18	75.96	1046.83	78.48	3570.11	713.46	68.15
PUFA	265.76	139.73	607.29	79.13	29.77	266.73	114.32	439.52	72.54	27.20

SD, standard deviation; FA, fatty acid; SFA, saturated fatty acid; MUFA, monounsaturated fatty acid; PUFA, polyunsaturated fatty acid.

**Table 3:** Spectral treatments and factors included in the prediction models of phospholipid fraction of fatty acids of beef.

Fatty Acid	Baseline Correction	Spectra Normalization	Scatter Correction ^a^	Smooth	Mathematical Treatment ^b^	F ^c^
12:0	Offset	None	EMSC	None	None	6
14:0	Offset	None	EMSC	None	SG-1-2-5	5
16:0	Offset	Area	SNV+D	None	SG-1-2-3	4
16:0 ald	None	None	None	None	SG-1-2-3	3
16:1	Offset	None	None	None	None	4
18:0	Offset	Area	EMSC	None	None	4
18:0 ald	Offset	Area	SNV+D	None	None	6
18:1 t9	None	None	EMSC	None	None	3
18:1 c9	None	None	None	None	SG-1-2-5	2
18:1 c11	Offset	Area	SNV+D	None	None	3
18:2 n-6	None	None	None	None	None	8
20:1	Offset	Area	SNV	None	None	6
18:3 n-3	Offset	None	None	None	None	10
18:2 9c11t CLA	Offset	Area	SNV+D	None	None	5
20:3 n-6	None	None	EMSC	None	None	3
20:4 n-6	None	Area	SNV	None	None	12
20:5 n-3	Offset	Area	EMSC+D	None	None	8
22:4 n-6	None	None	None	None	SG-1-2-3	5
22:5 n-3	Offset	Area	None	None	None	10
22:6 n-3	None	Area	None	None	None	1
Total FA	Offset	Area	None	None	SG-1-2-5	5
SFA	Offset	None	SNV	None	None	6
MUFA	None	None	SNV	None	None	5
PUFA	Offset	Area	None	None	SG-1-2-3	5

^a^ EMSC, extended multiplicative scatter correction; MSC, multiplicative scatter correction; SNV, standard normal variate; D, detrending. ^b^ SG, Savitzky–Golay derivative—derivative order—polynomial order—smoothing points; NG, Norris Gap derivative—derivative order—gap size. ^c^ F, number of factors.

**Table 4:** Spectral treatments and factors included in the prediction models of total fatty acids of beef.

Fatty Acid	Baseline Correction	Spectra Normalization	Scatter Correction ^a^	Smooth	Mathematical Treatment ^b^	F ^c^
12:0	None	None	SNV+D	None	SG-1-2-3	4
14:0	None	Area	SNV+D	None	SG-1-2-3	3
16:0	None	Area	SNV+D	None	SG-1-2-3	3
16ald	None	None	None	None	None	2
16:1	None	None	None	SG1-1-1	None	8
18:0	None	None	None	None	None	5
18ald	None	None	SNV+D	None	None	8
18:1t9	None	None	SNV+D	None	None	2
9c18:1	None	None	None	None	NG-1-13	7
11c18:1	None	Area	SNV+D	None	None	11
18:2n-6	None	None	None	SG1-2-2	SG-1-2-3	5
20:1	None	None	None	None	SG-1-2-3	4
18:3n-3	Offset	None	None	None	None	9
9c11tCLA	None	None	MSC	None	SG-1-2-3	4
20:3n-6	None	None	None	None	NG-1-7	7
20:4n-6	None	None	None	None	SG-1-2-3	7
20:5n-3	None	None	None	None	NG-1-15	7
22:4n-6	None	None	None	None	SG-1-2-3	6
22:5n-3	None	None	SNV	None	NG-1-7	5
22:6n-3	None	None	None	None	None	1
TotalFA	Offset	None	None	None	None	9
SFA	None	None	None	None	None	9
MUFA	None	None	None	None	NG-1-27	7
PUFA	None	None	None	None	NG-1-11	6

^a^ EMSC, extended multiplicative scatter correction; MSC, multiplicative scatter correction; SNV, standard normal variate; D, detrending. ^b^ SG, Savitzky–Golay derivative—derivative order—polynomial order—smoothing points; NG, Norris Gap derivative—derivative order—gap size. ^c^ Number of factors included in the calibrations.

**Table 5:** Calibration and validation statistics for the phospholipids fraction of fatty acid composition (mg FA/100 g of meat).

Fatty Acid	n	SEC	R^2^_c_	SEP	R^2^_P_	RPD	Consistency
12:0	206	0.04	0.48	0.15	0.07	1.06	26.67
14:0	215	0.46	0.77	0.73	0.52	1.44	63.01
16:0	210	7.52	0.67	10.45	0.48	1.43	71.96
16:0ald	210	5.31	0.24	6.70	0.13	1.08	79.25
16:1	205	1.35	0.44	1.85	0.32	1.24	72.97
18:0	211	5.72	0.50	6.92	0.36	1.27	82.66
18:0ald	210	3.10	0.40	5.17	0.10	1.06	59.96
t918:1	200	1.56	0.19	2.89	0.03	1.01	53.98
9c18:1	205	14.91	0.50	18.81	0.37	1.29	79.27
11c18:1	212	2.66	0.20	3.51	0.09	1.05	75.78
18:2n-6	200	18.81	0.42	33.94	0.04	0.87	55.42
20:1	215	0.15	0.49	0.20	0.27	1.16	75.00
18:3n-3	207	2.26	0.69	3.11	0.53	1.46	72.67
9c11tCLA	200	0.25	0.36	0.43	0.06	1.04	58.14
20:3n-6	201	1.56	0.26	2.08	0.11	1.07	75.00
20:4n-6	201	5.88	0.55	7.59	0.29	1.28	77.47
20:5n-3	202	1.34	0.56	1.74	0.41	1.30	77.01
22:4n-6	207	1.10	0.59	1.58	0.29	1.23	69.62
22:5n-3	200	1.73	0.58	2.47	0.41	1.33	70.04
22:6n-3	198	0.28	0.12	0.38	0.05	1.07	73.68
Total FA	199	39.14	0.67	63.88	0.44	1.40	61.27
SFA	202	14.80	0.65	20.68	0.57	1.50	71.57
MUFA	210	17.46	0.53	18.85	0.50	1.50	92.63
PUFA	198	19.77	0.65	31.84	0.14	1.24	62.09

n, number of samples used in validation; SEC, standard error of calibration; R^2^c, coefficient of determination of calibration; SEP, standard error of validation; R^2^p, coefficient of determination of validation; RPD = SD/SEP; Consistency (%) = SEC*100/SEP.

**Table 6:** NIRS calibration and validation statistics for the total fatty acid composition (mg FA/100 g of meat).

Fatty Acid	n	SEC	R^2^_c_	SEP	R^2^_p_	RPD	Consistency
12:0	222	1.02	0.70	0.97	0.72	1.9	105.15
14:0	219	26.05	0.77	27.97	0.74	2.0	93.14
16:0	222	233.56	0.71	234.99	0.72	1.9	99.39
16:0ald	222	5.98	0.13	6.66	0.16	1.0	89.79
16:1	218	32.17	0.76	30.31	0.78	2.1	106.14
18:0	222	158.17	0.61	129.39	0.70	1.8	122.24
18:0ald	222	3.79	0.53	4.85	0.21	1.1	78.14
t918:1	219	41.01	0.47	50.16	0.47	1.4	81.76
9c18:1	217	244.72	0.80	274.92	0.77	2.1	88.59
11c18:1	218	12.14	0.77	12.69	0.74	2.0	95.67
18:2n-6	222	41.63	0.56	41.32	0.43	1.3	100.75
20:1	220	1.40	0.80	1.10	0.71	1.8	127.27
18:3n-3	222	7.73	0.60	7.07	0.65	1.7	109.34
9c11t CLA	222	4.00	0.61	3.25	0.62	1.6	123.08
20:3n-6	217	1.45	0.64	2.09	0.39	1.3	69.38
20:4n-6	222	4.89	0.74	8.60	0.26	1.2	56.86
20:5n-3	210	1.71	0.39	1.90	0.38	1.3	90.00
22:4n-6	217	1.00	0.78	1.74	0.39	1.3	57.47
22:5n-3	211	2.47	0.37	2.79	0.36	1.2	88.53
22:6n-3	222	0.61	0.01	0.60	0.01	1.0	101.67
Total FA	222	908.22	0.74	730.79	0.79	2.2	124.28
SFA	222	412.56	0.73	355.68	0.77	2.1	115.99
MUFA	222	393.64	0.77	340.36	0.77	2.1	115.65
PUFA	222	53.35	0.54	53.81	0.45	1.3	99.15

n, number of samples used in validation; SEC, standard error of calibration; R^2^c, coefficient of determination of calibration; SEP, standard error of validation; R^2^p, coefficient of determination of validation; RPD = SD/SEP; Consistency (%) = SEC*100/SEP.

## Data Availability

Data presented in this study are available on request due from the corresponding author. The data are not publicly available due to privacy.

## References

[1] Denoyelle C., Cartier P. (1996). Measurement of ground beef composition by near infrared spectrophotometry. Viandes Prod. Carnes.

[2] Prieto N., Andres S., Giraldez F.J., Mantecon A.R., Lavin P. (2006). Potential use of near infrared reflectance spectroscopy (NIRS) for the estimation of chemical composition of oxen meat samples. Meat Sci..

[3] Olivan M., de la Roza B., Mocha M., Martinez M.J. Prediction of physico-chemical and texture characteristics of beef by near infrared transmittance spectroscopy. Proceedings of the 10th International Conference on Near Infrared Spectroscopy.

[4] Ripoll G., Lobon S., Joy M. (2018). Use of visible and near infrared reflectance spectra to predict lipid peroxidation of light lamb meat and discriminate dam’s feeding systems. Meat Sci..

[5] Ripoll G., Albertí P., Panea B., Olleta J.L., Sañudo C. (2008). Near-infrared reflectance spectroscopy for predicting chemical, instrumental and sensory quality of beef. Meat Sci..

[6] Aït-Kaddour A., Andueza D., Dubost A., Roger J.-M., Hocquette J.-F., Listrat A. (2020). Visible and Near-Infrared Multispectral Features in Conjunction with Artificial Neural Network and Partial Least Squares for Predicting Biochemical and Micro-Structural Features of Beef Muscles. Foods.

[7] Williams C.M. (2000). Dietary fatty acids and human health. Ann. Zootech..

[8] Galli C., Calder P.C. (2009). Effects of fat and fatty acid intake on inflammatory and immune responses: A critical review. Ann. Nutr. Metab..

[9] Enser M., Richardson R., Wood J., Gill B., Sheard P. (2000). Feeding linseed to increase the n-3 PUFA of pork: Fatty acid composition of muscle, adipose tissue, liver and sausages. Meat Sci..

[10] De Souza R.J., Mente A., Maroleanu A., Cozma A.I., Ha V., Kishibe T., Uleryk E., Budylowski P., Schünemann H., Beyene J. (2015). Intake of saturated and trans unsaturated fatty acids and risk of all cause mortality, cardiovascular disease, and type 2 diabetes: Systematic review and meta-analysis of observational studies. Bmj.

[11] Elmore J.S., Cooper S.L., Enser M., Mottram D.S., Sinclair L.A., Wilkinson R.G., Wood J.D. (2005). Dietary manipulation of fatty acid composition in lamb meat and its effect on the volatile aroma compounds of grilled lamb. Meat Sci..

[12] Mottram D.S. (1998). Flavour formation in meat and meat products: A review. Food Chem..

[13] Warren H.E., Scollan N.D., Nute G.R., Hughes S.I., Wood J.D., Richardson R.I. (2008). Effects of breed and a concentrate or grass silage diet on beef quality in cattle of 3 ages. II: Meat stability and flavour. Meat Sci..

[14] Mourot B.P., Gruffat D., Durand D., Chesneau G., Prache S., Mairesse G., Andueza D. (2014). New approach to improve the calibration of main fatty acids by near-infrared reflectance spectroscopy in ruminant meat. Anim. Prod. Sci..

[15] Prieto N., Dugan M.E., Lopez-Campos O., Aalhus J.L., Uttaro B. (2013). At line prediction of PUFA and biohydrogenation intermediates in perirenal and subcutaneous fat from cattle fed sunflower or flaxseed by near infrared spectroscopy. Meat Sci..

[16] Pullanagari R.R., Yule I.J., Agnew M. (2015). On-line prediction of lamb fatty acid composition by visible near infrared spectroscopy. Meat Sci..

[17] Andueza D., Listrat A., Durand D., Normand J., Mourot B.P., Gruffat D. (2019). Prediction of beef meat fatty acid composition by visible-near-infrared spectroscopy was improved by preliminary freeze-drying. Meat Sci..

[18] Blanco M., Peguero A. (2007). Determination of organic additives in mortars by near-IR spectroscopy. A novel approach to designing a sample set with high-variability components. Anal. Bioanal. Chem..

[19] Núñez-Sánchez N., Serradilla J., Ares J., Garrido-Varo A. (2008). Effect of moisture uptake on the repeatability of near infrared spectroscopy analyses of ewe milk using the dry extract system for infrared (DESIR) method. J. Near Infrared Spectrosc..

[20] Maeda H., Ozaki Y., Tanaka M., Hayashi N., Kojima T. (1995). Near Infrared Spectroscopy and Chemometrics Studies of Temperature-Dependent Spectral Variations of Water: Relationship between Spectral Changes and Hydrogen Bonds. J. Near Infrared Spectrosc..

[21] Coppa M., Ferlay A., Leroux C., Jestin M., Chilliard Y., Martin B., Andueza D. (2010). Prediction of milk fatty acid composition by near infrared reflectance spectroscopy. Int. Dairy J..

[22] Meurens M., Eydne O., Vanbelle M., Hollow J., Kaffka K.J., Gonczy J.L. (1987). Fine Analysis of Liquids by NIR Reflectance Spectroscopy of Dry Extract on Solid Support (DESIR).

[23] Ripoll G., Casasús I., Joy M., Molino F., Blanco M. (2015). Fat color and reflectance spectra to evaluate the β-carotene, lutein and α-tocopherol in the plasma of bovines finished on meadows or on a dry total mixed ration. Anim. Feed Sci. Technol..

[24] Giaretta E., Mordenti A., Palmonari A., Brogna N., Canestrari G., Belloni P., Cavallini D., Mammi L., Cabbri R., Formigoni A. (2019). NIRs calibration models for chemical composition and fatty acid families of raw and freeze-dried beef: A comparison. J. Food Compos. Anal..

[25] Beak S.H., Lee Y., Lee E.B., Kim K.H., Kim J.G., Bok J.D., Kang S.K. (2019). Study on the fatty acid profile of phospholipid and neutral lipid in Hanwoo beef and their relationship to genetic variation. J. Anim. Sci. Technol..

[26] Bourre J. (2005). Where to find omega-3 fatty acids and how feeding animals with diet enriched in omega-3 fatty acids to increase nutritional value of derived products for human: What is actually useful. J. Nutr. Health Aging.

[27] Scollan N.D., Dannenberger D., Nuernberg K., Richardson I., MacKintosh S., Hocquette J.F., Moloney A.P. (2014). Enhancing the nutritional and health value of beef lipids and their relationship with meat quality. Meat Sci..

[28] Blesso C.N. (2015). Egg phospholipids and cardiovascular health. Nutrients.

[29] Gandemer G. (1997). Lipides du muscle et qualité de la viande. Phospholipides et flaveur. OCL. OléagineuxCorps GrasLipides.

[30] European Union (2010). Directive 2010/63/EU of the European Parliament and of the Council of 22 September 2010 on the protection of animals used for scientific purposes. Off. J. Eur. Union.

[31] Albertí P., Panea B., Sañudo C., Olleta J.L., Ripoll G., Ertbjerg P., Christensen M., Gigli S., Failla S., Concetti S. (2008). Live weight, body size and carcass characteristics of young bulls of fifteen European breeds. Livest. Sci..

[32] Folch J., Lees M., Stanley G. (1957). A simple method for the isolation and purification of lipids from animal tissues. J. Biol. Chem..

[33] Scollan N.D., Choi N.-J., Kurt E., Fisher A.V., Enser M., Wood J.D. (2001). Manipulating the fatty acid composition of muscle and adipose tissue in beef cattle. Br. J. Nutr..

[34] Geesink G.H., Schreutelkamp F.H., Frankhuizen R., Vedder H.W., Faber N.M., Kranen R.W., Gerritzen M.A. (2003). Prediction of pork quality attributes from near infrared reflectance spectra. Meat Sci..

[35] Barlocco N., Vadell A., Ballesteros F., Galietta G., Cozzolino D. (2006). Predicting intramuscular fat, moisture and Warner-Bratzler shear force in pork muscle using near infrared reflectance spectroscopy. Anim. Sci..

[36] Williams P., Norris K. (1987). Near-Infrared Technology in the Agricultural and Food Industries.

[37] MacKintosh S.B., Richardson I., Kim E.J., Dannenberger D., Coulmier D., Scollan N.D. (2017). Addition of an extract of lucerne (Medicago sativa L.) to cattle diets–Effects on fatty acid profile, meat quality and eating quality of the M. longissimus muscle. Meat Sci..

[38] Wood J.D., Enser M., Fisher A.V., Nute G.R., Sheard P.R., Richardson R.I., Hughes S.I., Whittington F.M. (2008). Fat deposition, fatty acid composition and meat quality: A review. Meat Sci..

[39] Sevane N., Nute G., Sañudo C., Cortes O., Cañon J., Williams J.L., Dunner S. (2014). Muscle lipid composition in bulls from 15 european breeds. Livest. Sci..

[40] Sevane N., Levéziel H., Nute G.R., Sañudo C., Valentini A., Williams J., Dunner S. (2014). Phenotypic and genotypic background underlying variations in fatty acid composition and sensory parameters in European bovine breeds. J. Anim. Sci. Biotechnol..

[41] Khan M., Khan M.I., Sahar A., Jamil A. (2020). Predicting authenticity and physicochemical characteristics of meat through FT-IR spectroscopy coupled with multivariate analysis. Pak. J. Agric. Sci..

[42] Warren H.E., Scollan N.D., Enser M., Hughes S.I., Richardson R.I., Wood J.D. (2008). Effects of breed and a concentrate or grass silage diet on beef quality in cattle of 3 ages. I: Animal performance, carcass quality and muscle fatty acid composition. Meat Sci..

[43] Lucarini M., Durazzo A., Sanchez Del Pulgar J., Gabrielli P., Lombardi-Boccia G. (2018). Determination of fatty acid content in meat and meat products: The FTIR-ATR approach. Food Chem.

[44] Siemens B.J., Daun J.K. (2005). Determination of the fatty acid composition of canola, flax, and solin by near-infrared spectroscopy. J. Am. Oil Chem. Soc..

[45] Tøgersen G., Arnesen J., Nilsen B., Hildrum K. (2003). On-line prediction of chemical composition of semi-frozen ground beef by non-invasive NIR spectroscopy. Meat Sci..

[46] Cozzolino D., Murray I. (2002). Effect of sample presentation and animal muscle species on the analysis of meat by near infrared reflectance spectroscopy. J. Near Infrared Spectrosc..

[47] Tao F., Ngadi M. (2018). Recent advances in rapid and nondestructive determination of fat content and fatty acids composition of muscle foods. Crit. Rev. Food Sci. Nutr..

[48] Zhou L.J., Wu H., Li J.T., Wang Z.Y., Zhang L.Y. (2012). Determination of fatty acids in broiler breast meat by near-infrared reflectance spectroscopy. Meat Sci..

[49] Ortiz A., Parrini S., Tejerina D., Pinto de Araújo J.P., Čandek-Potokar M., Crovetti A., Garcia-Casco J.M., González J., Hernández-García F.I., Karolyi D. (2020). Potential Use of Near-Infrared Spectroscopy to Predict Fatty Acid Profile of Meat from Different European Autochthonous Pig Breeds. Appl. Sci..

[50] Pieszczek L., Czarnik-Matusewicz H., Daszykowski M. (2018). Identification of ground meat species using near-infrared spectroscopy and class modeling techniques—Aspects of optimization and validation using a one-class classification model. Meat Sci..

[51] Prieto N., Lopez-Campos O., Aalhus J.L., Dugan M.E., Juarez M., Uttaro B. (2014). Use of near infrared spectroscopy for estimating meat chemical composition, quality traits and fatty acid content from cattle fed sunflower or flaxseed. Meat Sci..

[52] Davies A.M.C., Grant A. (1987). Review: Near-Infrared analysis of food. Int. J. Food Sci. Technol..

[53] Sierra V., Aldai N., Castro P., Osoro K., Coto-Montes A., Olivan M. (2008). Prediction of the fatty acid composition of beef by near infrared transmittance spectroscopy. Meat Sci..

[54] Cecchinato A., De Marchi M., Penasa M., Casellas J., Schiavon S., Bittante G. (2012). Genetic analysis of beef fatty acid composition predicted by near-infrared spectroscopy. J. Anim. Sci..

[55] Zomeño C., Juste V., Hernandez P. (2012). Application of NIRS for predicting fatty acids in intramuscular fat of rabbit. Meat Sci..

[56] Hargrave-Barnes K.M., Azain M.J., Miner J.L. (2008). Conjugated linoleic acid-induced fat loss dependence on Delta6-desaturase or cyclooxygenase. Obesity.

[57] Cruz-Hernandez C., Deng Z., Zhou J., Hill A.R., Yurawecz M.P., Delmonte P., Mossoba M.M., Dugan M.E., Kramer J.K. (2004). Methods for analysis of conjugated linoleic acids and trans-18: 1 isomers in dairy fats by using a combination of gas chromatography, silver-ion thin-layer chromatography/gas chromatography, and silver-ion liquid chromatography. J. AOAC Int..

[58] Scollan N., Hocquette J.F., Nuernberg K., Dannenberger D., Richardson I., Moloney A. (2006). Innovations in beef production systems that enhance the nutritional and health value of beef lipids and their relationship with meat quality. Meat Sci..

[59] Barragan-Hernandez W., Mahecha-Ledesma L., Burgos-Paz W., Olivera-Angel M., Angulo-Arizala J. (2020). Using near-infrared spectroscopy to determine intramuscular fat and fatty acids of beef applying different prediction approaches. J. Anim Sci..

[60] Sato T., Kawano S., Iwamoto M. (1991). Near infrared spectral patterns of fatty acid analysis from fats and oils. J. Am. Oil Chem. Soc..

[61] Balabin R.M., Lomakina E.I. (2011). Support vector machine regression (SVR/LS-SVM)--an alternative to neural networks (ANN) for analytical chemistry? Comparison of nonlinear methods on near infrared (NIR) spectroscopy data. Analyst.

[62] Geladi P., MacDougall D., Martens H. (1985). Linearization and scatter-correction for near-infrared reflectance spectra of meat. Appl. Spectrosc.

